# Microfluidic-Derived Detection of Protein-Facilitated Copper Flux Across Lipid Membranes

**DOI:** 10.1021/acs.analchem.2c02081

**Published:** 2022-08-15

**Authors:** Kamil Górecki, Jesper S. Hansen, Ping Li, Niloofar Nayeri, Karin Lindkvist-Petersson, Pontus Gourdon

**Affiliations:** † Department of Experimental Medical Science, Faculty of Medicine, Lund University, Lund SE-22100, Sweden; ‡ Department of Biomedical Sciences, Faculty of Health and Medical Sciences, University of Copenhagen, Copenhagen N DK-2200, Denmark

## Abstract

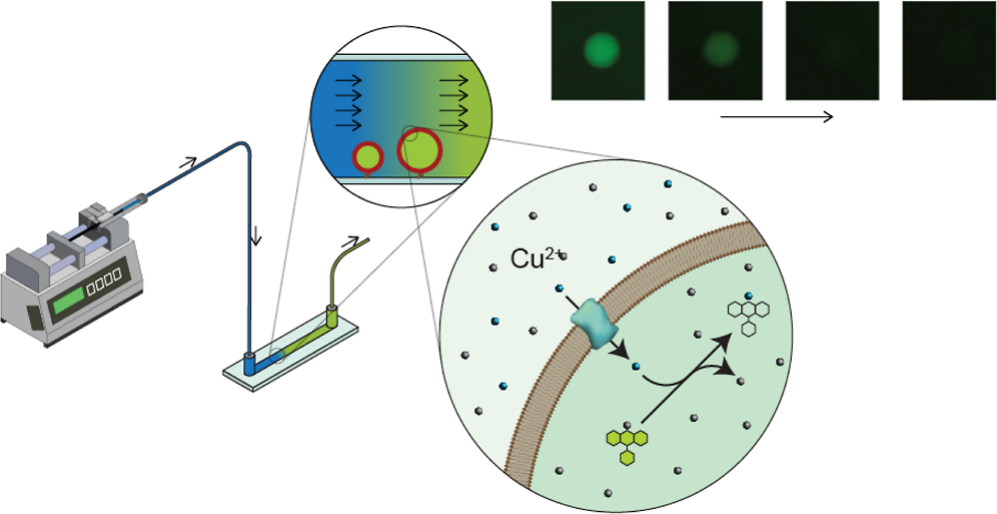

Measurement of protein-facilitated copper flux across biological membranes is a considerable challenge. Here, we demonstrate a straightforward microfluidic-derived approach for visualization and measurement of membranous Cu flux. Giant unilamellar vesicles, reconstituted with the membrane protein of interest, are prepared, surface-immobilized, and assessed using a novel quencher–sensor reporter system for detection of copper. With the aid of a syringe pump, the external buffer is exchanged, enabling consistent and precise exchange of solutes, without causing vesicle rupture or uneven local metal concentrations brought about by rapid mixing. This approach bypasses common issues encountered when studying heavy metal-ion flux, thereby providing a new platform for *in vitro* studies of metal homeostasis aspects that are critical for all cells, health, and disease.

## Introduction

Heavy metals such as zinc (Zn) and copper (Cu) are essential for all living organisms, from bacteria to mammals. Cu is critical as a co-factor in numerous proteins, including enzymes involved in redox reactions and oxygen transport.^1^ Due to its toxicity at elevated concentrations, Cu levels however need to be tightly regulated. In addition, Cu is used both as a natural biocide^1,2^ and also as antimicrobial agent in medicine.^3^

In living cells, the homeostasis is regulated by a sophisticated balance between import and export processes and by intracellular ‘buffering’ (sequestering) of the available metal. Transmembranous regulation is maintained by specialized membrane protein heavy metal transporters and channels. As an example, malfunctioning of the human copper transporters ATP7A and ATP7B is directly linked to the severe Menkes’ and Wilson’s diseases,^4^ respectively, indicative of the biological significance of aberrant copper homeostasis. Consequently, a detailed understanding of the fundamental molecular principles that govern these proteins is essential to shed further light on their physiological role and possibly also to manipulate the metal homeostasis.^19,20,22^

Heavy metal-ion flux has often been measured either in living cells expressing the protein of interest, followed by measurement of concentration changes over time,^5−7^ or by reconstitution of the protein of interest into vesicles, typically with a fluorescent reporter dye trapped inside, allowing measurement of the metal-ion concentration.^8−10^ Giant vesicles offer a synthetic cell-like system in terms of curvature and membrane fluid dynamics. Although still limited, giant unilamellar vesicles (GUVs) have been successfully used in studies of membrane protein-mediated transport, including for K^+^ channels, solute carriers, GPCRs, proton pumps, and aquaporins.^11−13^ Heavy metal transport investigations have also been reported for a Cu^+^-transporting *P*-type ATPase, however, only by indirect measurements of metal transport measured by inductively coupled plasma mass spectrometry.^14^

A common challenge encountered when measuring heavy metal flux in artificial membrane setups is the rapid delivery of a high concentration of the metal ions. Hitherto, the reaction has been commenced by addition of a small volume of concentrated metal salt in solution and subsequent rapid mixing using a stop-flow cell. This approach has a limitation of requiring a fast and often turbulent mixing of the solutions, which may result in locally high concentrations of metal ions and in bursting of proteoliposomes. Alternatively, a slow addition of a solution with only slightly increased metal-ion concentration can be employed. However, this approach is associated with prolonged reaction time that can cause significant photobleaching of the reporter dyes and introduction of damage to the protein and the lipids. These difficulties may lead to incorrect conclusions and conflicting reports.^13,15^

**Figure 1:**
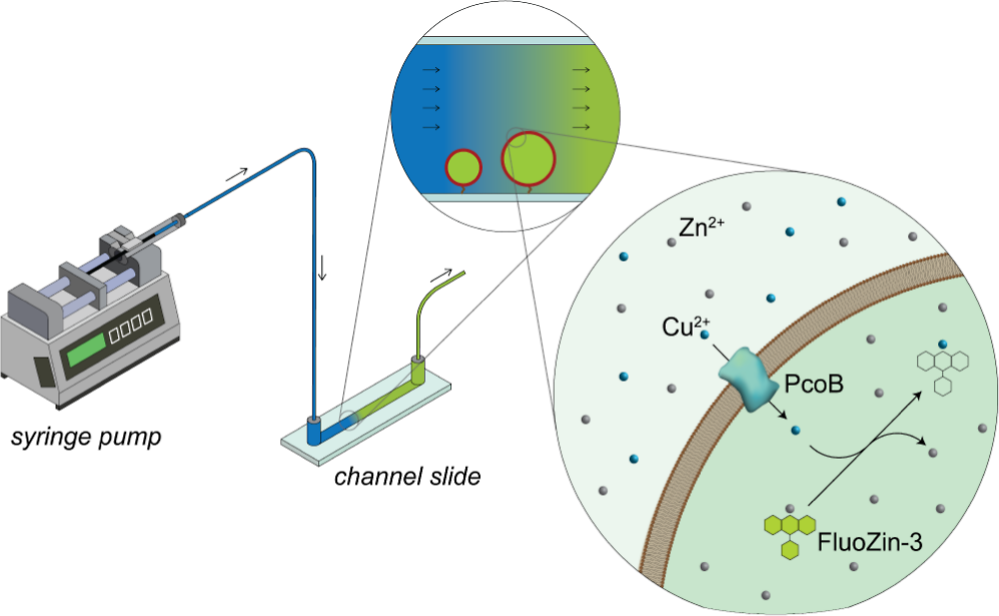
Schematic of the microfluidic setup for measurement of Cu flux in giant unilamellar vesicles. The three magnifications of the experiment are shown: the equipment used for the experiments, the process observed in the channel slides, and the molecular mechanism of the reactions.

Here, we present a setup that bypasses these challenges by combining the use of immobilized giant unilamellar vesicles (GUVs) with a microfluidic setup (Figure 1). Building on our previous successful experiences with reconstitution of membrane proteins into artificial vesicles, we employed the method to study copper flux in real time.^16−18^ As a proof of concept, we designed a setup combining a membrane protein metal-ion channel reconstituted into GUVs with a fluorescent sensor to detect Cu^2+^ flux. Specifically, we selected the outer membrane protein PcoB from *Escherichia coli* that serves as a Cu-specific porin in a number of bacteria.^5,19−21^ To follow the changes in the Cu^2+^ concentrations, we employed a membrane-impermeable Zn sensor FluoZin-3.^8^ In a complex with Zn^2+^ ions, FluoZin-3 exhibits strong and stable fluorescence, with 494 nm excitation and 516 nm emission maxima. This complex is however perturbed by Cu^2+^ ions, which bind with over 100 times higher affinity to FluoZin-3 than Zn^2+^ ions, thus providing a means to measure the concentration of Cu^2+^.^22^ Through encapsulation inside the GUVs, the dye was used as a Cu^2+^ indicator of the vesicular interior. Taken together, this setup enabled direct observation of protein-mediated heavy metal flux over lipid membranes.

## Experimental Procedures

### Materials

The lipids were purchased from Avanti Polar Lipids. FluoZin-3 tetrapotassium salt, Pacific Blue NHS, and Atto 488 NHS were purchased from Thermo Fisher. All other chemicals were purchased from Merck/Sigma Aldrich.

### Protein Production

A codon-optimized *E. coli* pcoB gene was synthesized (GenScript), cloned into a pET22 vector with a N-terminal His tag, and transformed into the C43 *E. coli* strain. 1 L culture in standard LB medium was grown at 37 °C and 200 rpm in 3 L baffled flasks to OD 0.6. Then, the temperature was lowered to 20 °C, and IPTG was added to 1 mM final concentration. After 20 h, the cells were harvested by centrifugation, washed in 50 mM phosphate buffer pH 7.4, and broken with an X-cell system for disruption of cells. Total membranes were collected by ultracentrifugation and resuspended in phosphate buffer. 1% (w/v) sarcosyl was used to dissolve the inner membrane, and subsequently, a 2% Elugent detergent mixture was used to solubilize the PcoB-containing outer membranes. The solubilized protein was captured on a HisTrap Ni-affinity column. The protein was eluted with 250 mM imidazole, and concurrently, a detergent exchange to 1% (w/v) octyl glucoside, OG, was achieved. The protein was subjected to Superdex 200 size exclusion chromatography, and the peak fraction was collected, concentrated to about 1 mg/mL, flash-frozen in liquid nitrogen, and kept at −80 °C until needed.

### Protein Labeling

The protein was labeled with Pacific Blue dye succinimidyl ester or with Atto 488 succinimidyl ester (Thermo Fisher Scientific) according to the manufacturer’s instructions. Briefly, the PcoB-containing solution in 0.1 M sodium bicarbonate buffer (pH 8.3) was mixed with dye solution in DMSO and incubated for 1 h at 18° C. Excess dye was then removed, and the buffer was exchanged to phosphate buffer with a spin column. The labeled protein was used in the preparation of GUVs.

### GUV Production

The method for GUV preparation was performed as previously described,^16^ with the following modifications. 1% (w/v) ultra-low-melting agarose was prepared in mQ water (10 μL) at 80 °C, and upon complete dissolving, it was allowed to cool down at 18 °C. Subsequently, about 1 μL of the protein solution was added (1 μg of total protein) to 10 μL of agarose gel. This protein–agarose gel was then deposited with a pipette on a coverslip plasma-etched with a handheld plasma treater (Electro-Technic Inc). In order to form a thin agarose film, another coverslip was dropped onto the first one and swiped over. The protein–agarose gel film was then allowed to dry for about 10 min at 18 °C.

As a basis for GUVs, 1,2-diphytanoyl-*sn*-glycero-3-phosphocholine, DPhPC, was used. The lipid was doped with 0.4 mol % rhodamine-labeled 1,2-dipalmitoyl-*sn*-glycero-3-phosphoethanolamine (DPPE) in order to visualize the membranes and with biotin-tagged DPPE in order to allow attachment to the slides via a biotin–streptavidin anchor. 10 μL of the lipid mixture (10 mM DPhPC, 0.1 mM DPPE-biotinyl, and 0.4% (w/v) rhodamine-DPPE, in CHCl_3_) was sprayed over the protein–agarose gel film under a stream of nitrogen (for details, see^23^). The coverslips were allowed to dry for additional 5 to 10 min.

The coverslip with the double film consisting of protein–agarose gel covered with lipids was placed inside a Sykes–Moore chamber and covered with 400 μL of swelling buffer; in order to avoid interference between buffers and metal ions, many commonly used buffers had to be avoided.^24^ In particular, Tris in combination with Zn under prolonged contact may cause unspecific PcoB degradation.^21^ We therefore chose 20 mM MOPS with pH adjusted to 7.4 with NaOH, unless mentioned otherwise. 150 mM NaCl or Na_2_SO_4_ and 15 mM KCl or K_2_SO_4_ were used throughout the experiments. The swelling buffer contained 50 μM FluoZin-3, 5 μM valinomycin, and 100 mM raffinose. Unless stated otherwise, 50 μM ZnSO_4_ was also included.

After approximately 45 min of swelling, 350 μL of the swelling mixture was withdrawn from the chambers with a wide-tip pipette and put into a test tube containing 1.5 ml of sinking buffer. The sinking buffer had the same composition as the swelling buffer in the corresponding experiment, with the exceptions of 100 mM sucrose used instead of raffinose, and no FluoZin-3 or valinomycin was present. As a modification to the previous work,^16^ glucose was avoided here as it reacts with Cu^2+^, being a reducing sugar. Due to the high density of the swelling (internal) buffer, the vesicles settled down at the bottom of the test tubes, thus increasing local concentration.

### Microscopy

**Figure 2:**
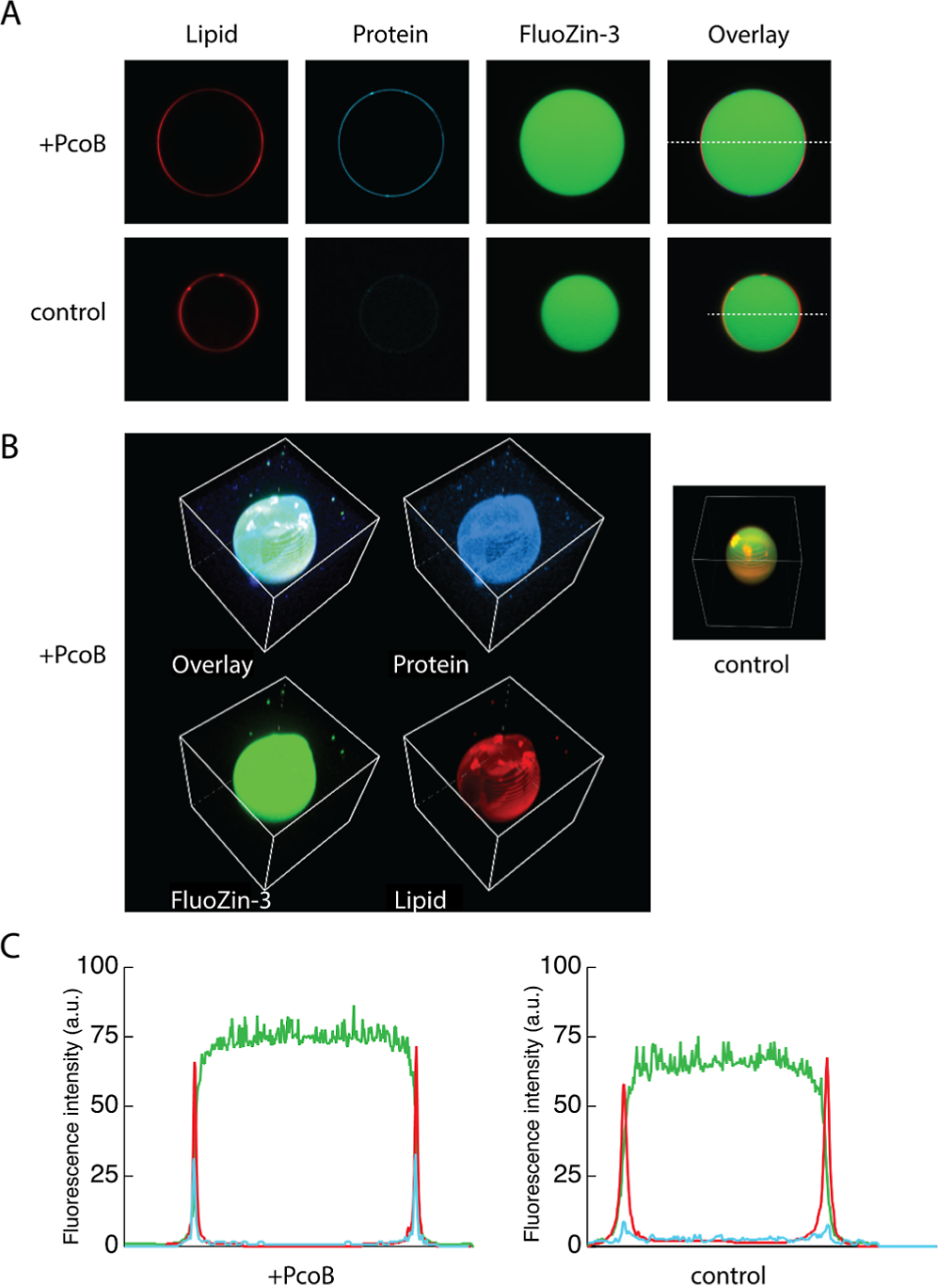
Micrographs of representative samples of GUVs, a PcoB-containing and a protein-free vesicles were chosen, and micrographs were recorded in three channels. (A) Slice of vesicles, showing the fluorescent images in three channels: rhodamine-labeled lipids (red), Pacific Blue-labeled PcoB (blue), and FluoZin-3-Zn complex (green). (B) Z-stack of a PcoB-containing vesicle showing a nearly perfect spherical shape, with the same colors as in panel A. (C)Intensity profiles of the PcoB-containing and control GUVs along the dashed lines in (A), respectively. The colors correspond to the fluorescent dyes.

The microscope used for the selection of the vesicles, the vesicle quality control, and conducting the experiment was Axiovert 200, and the images were captured with a AxioCam ICc 5 camera at intervals of 5 s. The experiments were observed with x5, x10, and x40 objectives. The micrographs presented in Figure 2 were recorded with a Nikon Confocal A1RHD microscope.

### Metal Flux Assay

The assay was performed on 0.5 mm-thick channel slides from Ibidi. The slides were first washed with mQ water and then coated with streptavidin (1 mg/mL in mQ water) for at least 30 min. The channels were then rinsed with water and filled with 40 μL of assay buffer containing no metal. 5–10 μL of the vesicle suspension in the sinking buffer withdrawn with a wide-tip pipette was added subsequently to the channels, which were then tilted slightly to allow for the vesicles to spread evenly inside the channel. The distribution of the vesicles was monitored with a microscope, and once a satisfactory distribution was achieved, the vesicles were allowed to settle down and attach via the biotin–streptavidin anchor by letting the channels incubate for least 10 min.

Once settled, the initial pictures were taken under a rhodamine filter, in order to record the starting point of the experiment. A number of GUVs were then chosen based on their shape, size, and lipid-label fluorescence intensity prior to flux measurements (in order to exclude multilamellar or faulty vesicles). Then, the filter was changed to FITC, and the exposure time was adjusted to give about 90% maximum. The reactions were started either by adding 0.5 μL of 1 M CuCl_2_ to one side of the channel slide in the case of manual delivery or by slowly pumping the Cu-containing buffer with the help of a syringe pump. The flow rate was chosen to not to influence the position or shape of the vesicles and to prevent mixing between the solutions while pumping (for further details, see Results and Discussion).

After no noticeable fluorescence was seen anymore (usually after 10 min in the case of syringe pump usage or 3–5 min when adding CuCl_2_ with a pipette), the experiment was concluded.

### Data Analysis

The pictures were exported as JPEGs with maximum quality, and the fluorescence signals were quantified using ImageJ (“total intensity”). Due to occasional residual FluoZin-3 outside of the vesicles, background values were subtracted using the same areas as corresponding vesicles, and the numbers were plotted against time. In cases where bleaching was significant, it was subtracted from the values. Data recorded from vesicles of similar diameters were pooled, and flux curves were obtained. See Figure S1 of the Supporting Information for details of image analysis.

## Results and Discussion

### GUV Production and Protein Incorporation

Among the variety of methods for reconstituting membrane proteins into GUVs, hydrogel-assisted swelling from partially dehydrated agarose gel is the most straightforward, requires no specialized equipment, and is sufficiently fast to avoid protein denaturation. This method has previously been successful for reconstitution of aquaporins,^25^ bacteriorhodopsin,^25^ GPCRs,^26^ and glucose transporters (GLUTs)^16^ and permitted assessment of protein-mediated transport of GLUT-containing GUVs.

We designed the current work based on the lessons learned from GLUTs. DPhPC was selected for preparation of GUVs. This synthetic derivative of phosphocholine forms stable bilayers of low permeability to ions and is less prone to oxidation compared to traditionally used lipids (e.g., 1-palmitoyl-2-oleoyl-*sn*-glycero-3-phosphocholine, POPC). To allow for attachment of the vesicles to the bottom of the channel slides, biotinylated DPPE was also added (to 1% total lipids), and in order to distinguish between unilamellar and multilamellar vesicles, the lipid mixture was doped with 0.4 mol % DPPE-rhodamine. Notably, addition of these two lipids was crucial for obtaining low permeability of the vesicles, perhaps due to the different nature of the DPPE head group, which allows for tighter arrangement of the lipid molecules in the bilayer, likely due to hydrogen bonding between the primary amine and either the headgroup phosphate or backbone carbonyls of neighboring lipids.^27^ This observation demonstrates the main advantage of our setup: an acceptable lipid–protein combination was easily identified due to the ability of direct observation of the behavior of individual vesicles.

A representation of typical high-quality vesicles obtained with our method is shown in Figure 2. Most vesicles were round and unilamellar, with diameter in the micrometer range (5–20 μm), as revealed by rhodamine detection (Figure 2A,B, in red).

To investigate the incorporation of PcoB into the lipid bilayer, the protein was labeled with Pacific Blue NHS ester, a lysine-reactive and UV-excitable fluorophore, prior to reconstitution (Figure 2A,B, in blue). As judged from the fluorescence intensity, insertion of the protein into the lipid bilayer was achieved with ease, and no optimization was needed. FluoZin-3 was clearly retained in the intravesicular compartment after washing with a buffer lacking FluoZin-3 (Figure 2A,B, in green). Linear profiles across vesicles (dashed lines in Figure 2A) showed two peaks of fluorescence coinciding with the side view of the membrane, and intra-vesicular spaces showed stable fluorescence, suggesting lack of artifacts in the formed membranes (Figure 2C). Vesicles not meeting these criteria were not used for analysis (see Figure S2 for an example of a faulty vesicle, Figure S3 for examples of multilamellar and unilamellar vesicles, and Figure S4 for an example of the variability of protein reconstitution in the vesicles).

### Dye Incorporation, Stability, and Membrane Permeability

**Figure 3:**
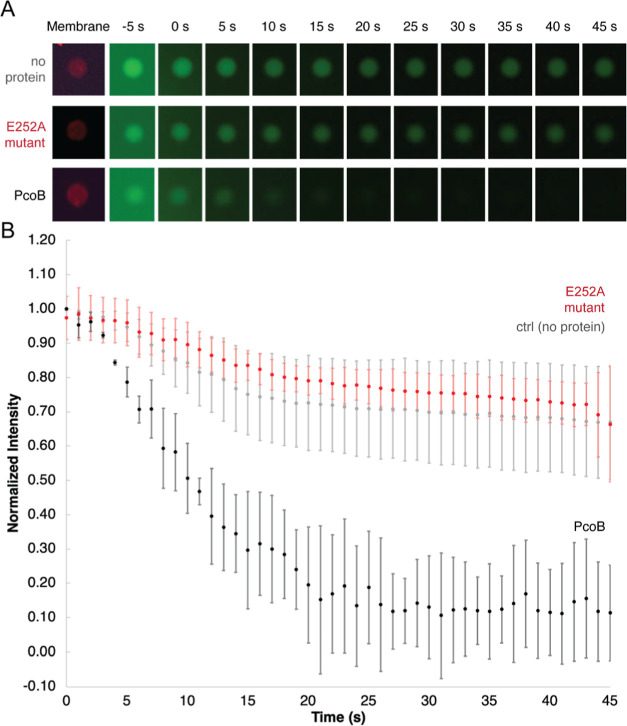
Flux curves for proteoliposomes subjected to copper delivered using a pipette. (A) Representative GUVs recorded in 5 s intervals for vesicles incorporated with wild-type or E252A PcoB or with empty GUVs as a control. The sizes of the vesicles were estimated to be 5–7 μm in diameter. (B) Time course of the Cu flux, 0.5 μL of 1 M CuCl_2_ was supplemented to one side of the channel slide. PcoB-containing GUVs (black circles, *n* = 3 vesicles from one representative experiment), PcoB mutant E252A-containing GUVs (red circles, *n* = 7), and control vesicles (gray, *n* = 8) are shown. Error bars represent standard deviations. The vesicles were from the same batch.

Control GUVs (without reconstituted protein) were practically impermeable within the time frame of the experiments (Figure 3). Under high magnification (over 40×) and for samples not measured immediately after preparation, some bleaching was observed; therefore, the light exposure was kept at a minimum even when using low magnifications. Alternatively, bleaching was subtracted from the flux curves (see Figure S1 for details). When measuring Cu flux, Zn^2+^ was present inside GUVs to provide the initial fluorescent signal. In order to preserve the high initial fluorescence, the same Zn^2+^ concentration was present in the sinking buffer used for the vesicles.

### Vesicle Quality

Similar to the results observed in other articles on GUVs,^28^ we noticed that some GUVs exhibited higher lipid-label fluorescence and that the profiles of these vesicles did not look as expected for unilamellar vesicles. Due to these defects, we deemed these GUVs to not be unilamellar, and they were therefore excluded from flux analysis.

### Cu Flux Assay Preparations

Immediately before the assays, the vesicles were allowed to attach to the bottom of the glass channels slides via the biotin–streptavidin anchor, and the external solution was exchanged to remove FluoZin-3. The anchoring ensured that the GUVs did not move during the measurement, which facilitated quantification and the verification of intactness once the measurements were completed.

### Initial Cu Flux with Manual Cu Delivery

We first investigated the Cu flux abilities of PcoB-containing GUVs by manually adding Cu salt solution to one side of the channel slide. This proved to be more difficult than expected. Adding a low concentration of CuCl_2_ required extensive time for diffusion of Cu ions to reach the vesicles (often over 30 min), and higher concentrations resulted in visible Cu precipitation in the buffer of near-neutral pH and subsequently an unknown final Cu concentration around the vesicles. We estimated the final internal Cu concentration to be at least 10 mM, given the visible precipitation; however, it was difficult to further improve this estimate. After a laborious period of testing, a suboptimal compromise between the speed of diffusion and signal-to-noise ratio was achieved (Figure 3 and Figure S5, with 1 M CuCl_2_). We could indeed observe a PcoB-mediated Cu influx, resulting in quenching of Fluozin-3:Zn complex fluorescence, whereas in protein-free GUVs, the fluorescence decreased only slightly. However, protein incorporation may cause a non-specific leakage due to insufficient interactions between the lipid and the proteins. In order to rule out this, we used a mutated version of PcoB as a control. The E252A PcoB form, with a conserved negatively charged glutamate residue in the ion path exchanged to an alanine, behaved as the protein-free control.^28^ Thus, it is likely that the Cu flux takes place through the pore of the protein in the wild-type form, while passage is limited to diffusion in GUVs without protein or when the mutant PcoB is incorporated. However, we could not conclude any further details regarding the transport due to the difficulties encountered with Cu delivery to the GUVs. Furthermore, the initial stages of the flux are clouded by the uneven mixing of the Cu with the surrounding buffer.

### Microfluidic Delivery of Cu

To improve the Cu delivery to the PcoB-containing GUVs, we decided to establish a microfluidic setup. We came up with a straightforward approach of connecting a syringe pump via Teflon tubing to a microfluidic chip, commercially available as six-channel microscope slides (Figure 1). Such slides are routinely employed in microbiological assays, for instance, for live cell imaging under flow. In a similar fashion, the vesicles are attached to the bottom of the channel via the biotin–streptavidin anchor. The additional advantage here, compared to using Sykes–Moore chambers, is the very low volumes needed for each experiment as the slides are of 0.4 mm height, resulting in the total volume needed for the experiment to be less than 50 μL.

A solution containing a 1000× lower concentration of Cu ions than in the previous setup was placed in the syringe and was slowly pumped through the channel slide to thereby avoid Cu precipitation issues. The sucrose used in the sinking buffer was exchanged to a lighter sugar, sorbitol, resulting in lower density of the Cu-containing buffer, yet with similar viscosity and osmotic strength. It may also prevent occurrence of uneven Cu concentrations while pumping. This adjustment ensured minimization of the mixing at the boundary of the two solutions, providing a clear start point for the flux reaction. Moreover, the final delivered Cu concentration was expected to be stable and controllable, in contrast to the manual Cu delivery.

Additionally, prior to measuring Cu flux, the buffer in the channel slides was completely exchanged by slowly pumping a Zn-free buffer through the channel, which resulted in virtually no background fluorescence. Moreover, a combination of degassing the buffers prior to usage and the closed system provided by the syringe pump, tubings, and the channel slide decreased the amount of dissolved oxygen and thus hindered the occurrence of photobleaching and likely also improved the stability of the proteins and lipids. This treatment likely improved the signal-to-noise ratio and enabled much longer reaction times without raising concerns about excessive damage to the samples.

**Figure 4:**
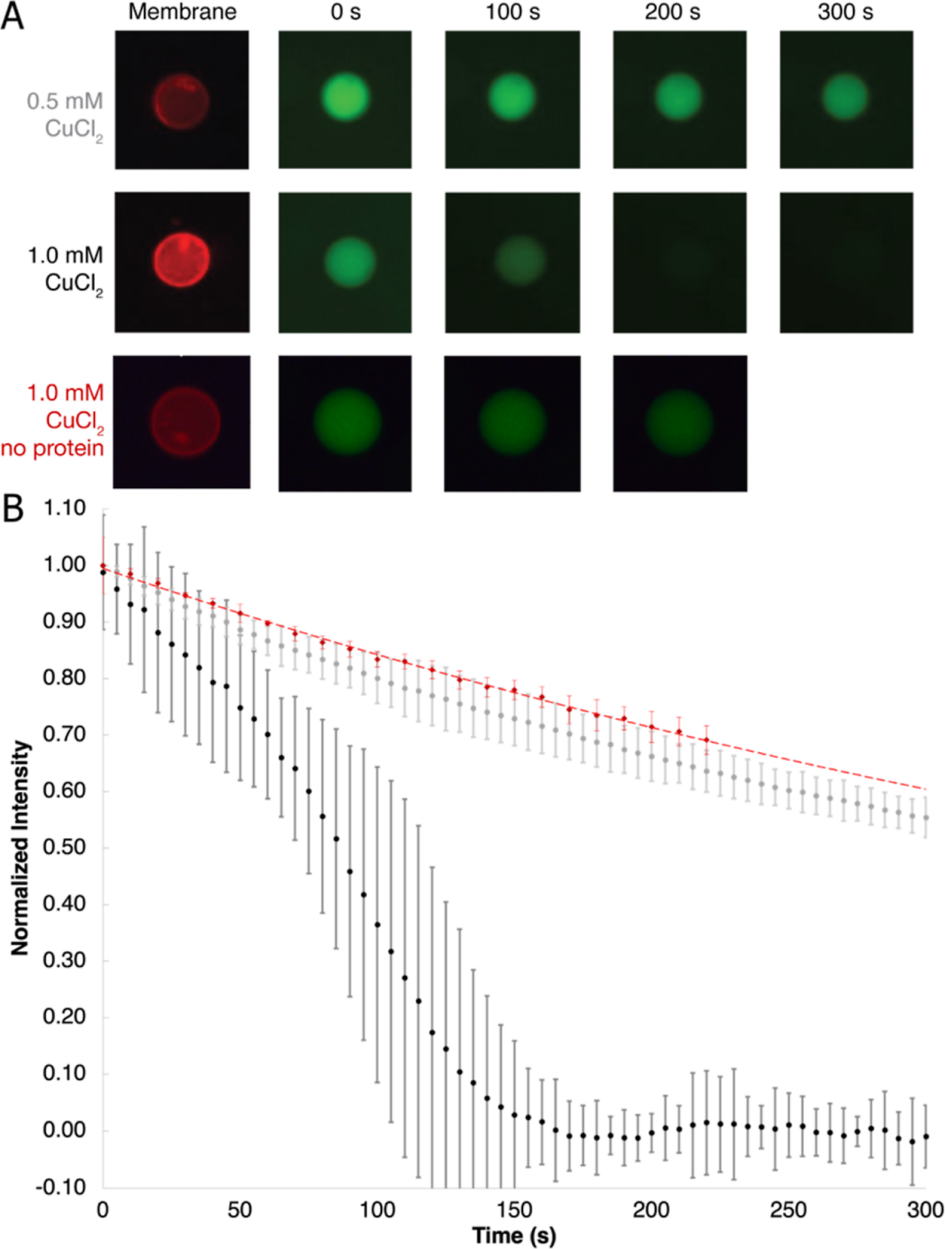
Flux curves for proteoliposomes subjected to copper delivered using a syringe pump. (A) Representative GUVs recorded in 100 s intervals for vesicles incorporated with wild-type PcoB. The shown vesicles were estimated to be about 5 μm in diameter. (B) Time course of the Cu flux, 1 mM (black circles, *n* = 5) or 0.5 mM (gray circles, *n* = 6) were supplemented to the GUVs. A control sample with 1 mM Cu and no protein reconstituted into GUVs is also shown (red circles, *n* = 4). Error bars represent standard deviations. The vesicles were from the same batch.

The microfluidic setup enabled reliable testing of different initial Cu concentrations (Figure 4 and Figure S6, with 1 mM CuCl_2_). As expected, for the passive flux typically associated with passage across outer membranes, higher initial concentration of the cargo caused a faster fluorescence decrease. Due to an overall slow flux and low background fluorescence, more data points could be collected during the reaction, resulting in a higher precision of the recorded flux curves. As evident when comparing Figures 3 and 4, the curves obtained with syringe pump-mediated Cu delivery were smoother. Taken together, the results presented here suggest that membrane proteins linked to Cu homeostasis can be studied using the presented method. The observation that PcoB conducts Cu^2+^ corroborates with its presented role in overall copper homeostasis in Gram-negative bacteria.

## Conclusions

We have demonstrated a successful reconstitution of a membrane protein related to heavy metal homeostasis into giant unilamellar vesicles by hydrogel-assisted swelling. This method allows direct investigation of metal transport functions, which was demonstrated with the pore-form outer membrane protein PcoB from *E. coli*.

## Supporting Information Available

Details on data handling and quality control (PDF)

## Abbreviations

DPhPC: 1,2-diphytanoyl-*sn*-glycero-3-phosphocholine
DPPE: 1,2-dipalmitoyl-*sn*-glycero-3-phosphoethanolamine
GLUTs: glucose transporters
GUV: giant unilamellar vesicle
ICP-MS: Inductively coupled plasma mass spectrometry
PcoB: plasmid-encoded copper resistance outer membrane protein.
