# Supplementary material for: Microfluidic-Derived Detection of Protein-Facilitated Copper Flux Across Lipid Membranes
Source: Anal Chem. 2022 Aug 15;94(34):11831–7. doi: 10.1021/acs.analchem.2c02081 (PMC9434548; doi:10.1021/acs.analchem.2c02081)
Supplement: Supplementary file 1 — ac2c02081_si_001.pdf [file ac2c02081_si_001.pdf]

Supporting information for

Microfluidic derived detection of protein facilitated copper flux across lipid membranes.

Kamil Górecki, Jesper S. Hansen†, Ping Li, Niloofar Nayeri, Karin Lindkvist-Petersson, Pontus Gourdon*.

Department of Experimental Medical Science, Faculty of Medicine, Lund University, SE-22 100 Lund, Sweden

Department of Biomedical Sciences, Faculty of Health and Medical Sciences, University of Copenhagen, DK-2200 Copenhagen N, Denmark

Data analysis

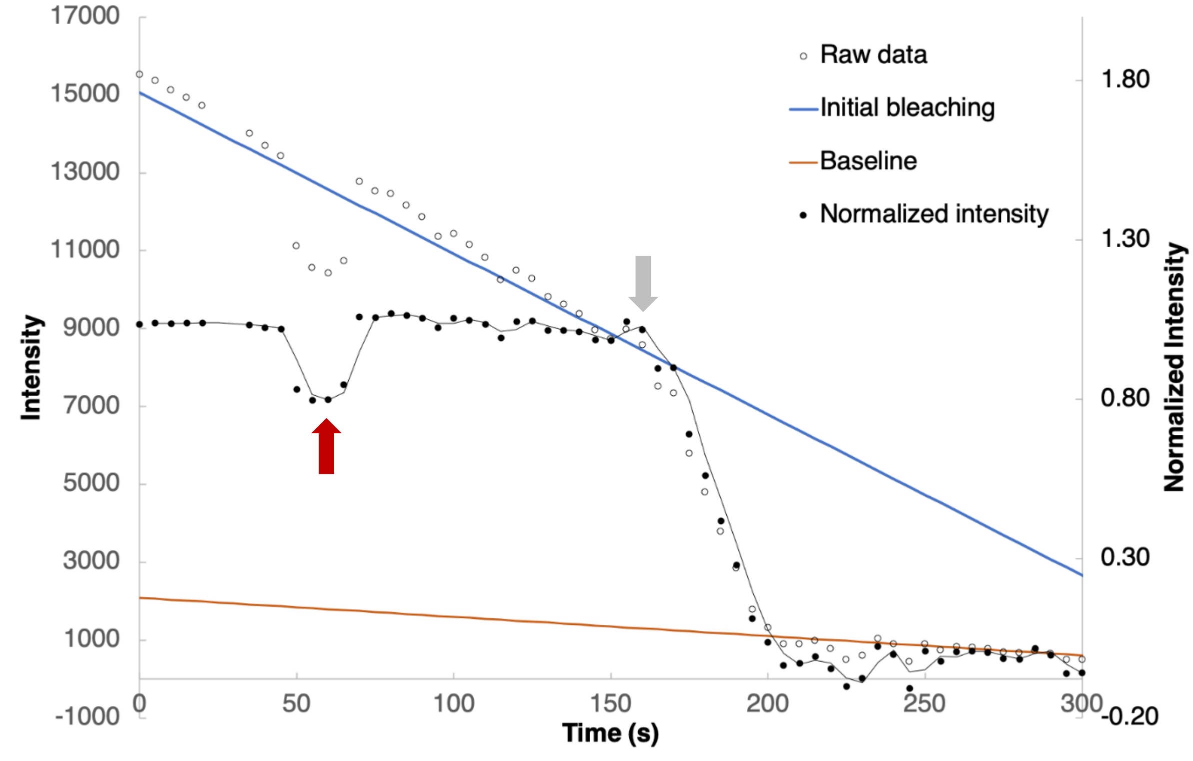

Figure S1. An example of data processing and analysis for a GUV exhibiting bleaching prior to measurements.

Fig. S1 shows an example of how normalization of recorded fluorescence was performed in cases where pre-measurement bleaching or unstable baseline was observed. The open circles represent measured fluorescence intensities and, as evident, there was a linear decrease in fluorescence even before addition of Cu. This behavior was observed for GUVs older than a few hours, and is likely related to increasing amounts of dissolved oxygen in the buffers. Other changes to the baseline were also observed (likely due to movement of the vesicles upon measurements). Therefore, a straight line fit to the points prior to Cu addition (blue, 0-150s) was subtracted from the measurements, as well as baseline calculated from the points after the Cu flux was done (200 s onwards). In cases when the fluorescence did not completely disappear, no post-measurement baseline was subtracted from the measurements. A moving average (black line) was used to pinpoint the starting point of the flux (gray arrow).

Occasionally, a few measurement points were clearly outside of the fit curves (red arrow). It was often caused by slight movement of vesicles (either to sides or out of focus), or uneven diffusion of Cu in the external solution, resulting in uneven background. Yet, we chose to not remove any data points, as that could potentially lead to bias.

Faulty vesicles

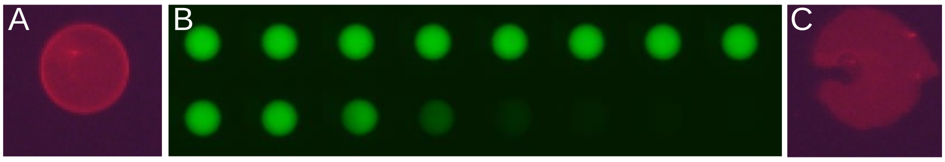

Figure S2. Burst vesicle. A, A GUV containing PcoB and its membrane visualized using rhodamine before the addition of Cu; B, Time course of fluorescence change of FluoZin-3-Zn complex after addition of Cu, images recorded in 5 s intervals, starting from 0 s; C, Membrane visualized following 80 s past addition of Cu. The vesicle size here was estimated to 6 μm.

The ability to visualize the rhodamine-stained membranes allows for identification of faulty vesicles. Here the Cu salt was added with a syringe pump. Note the fluorescence disappears rapidly (within a minute, most within 20 s), compared to the rather slow process in case of intact vesicles. We note removal of broken vesicles allowed for more accurate estimation of the Cu flux.

Selection of high-quality vesicles

The ability to visualize the rhodamine-stained membranes also allows for identification of patchy and multilamellar vesicles. As shown in Fig. S3, the preparation often contained vesicles of ranging fluorescence intensity. While upon observation of the FluoZin-3 signal, there is no difference between the vesicles. However, when observed under the rhodamine filter, the high fluorescence of some vesicles was obvious. Only the vesicles of the lowest fluorescence were chosen for measurements, and it was assumed that higher fluorescence intensity meant multilamellarity (vesicles marked with arrows).

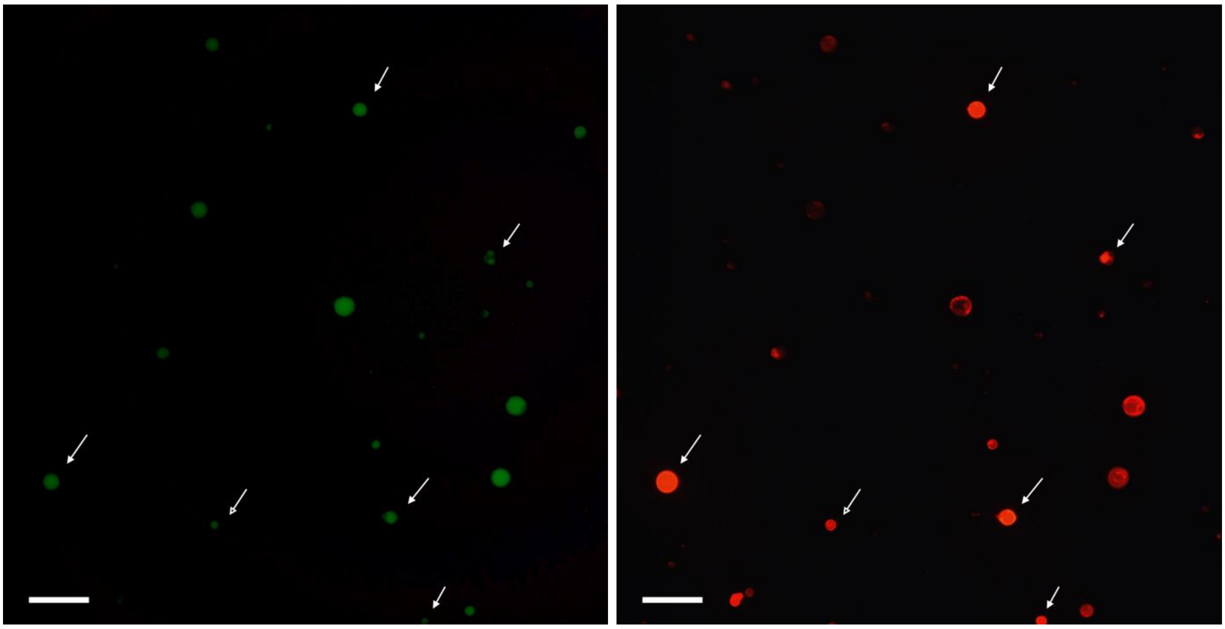

Figure S3. Multilamelar vesicles. Left, FluoZin-3 signal; right, rhodamine-signal. Vesicles judged not to be unilamellar are marked with arrows.

Protein incorporation

To investigate the variability of protein reconstitution in the vesicles, we labeled the protein with ATT488 NHS, and recorded the fluorescence under the FITC filter (note the FluoZin-3 was omitted in this preparation, as it has overlapping fluorescence spectrum with ATT488). Fig. S4 shows a low variation in protein incorporation, which suggests an optimal preparation.

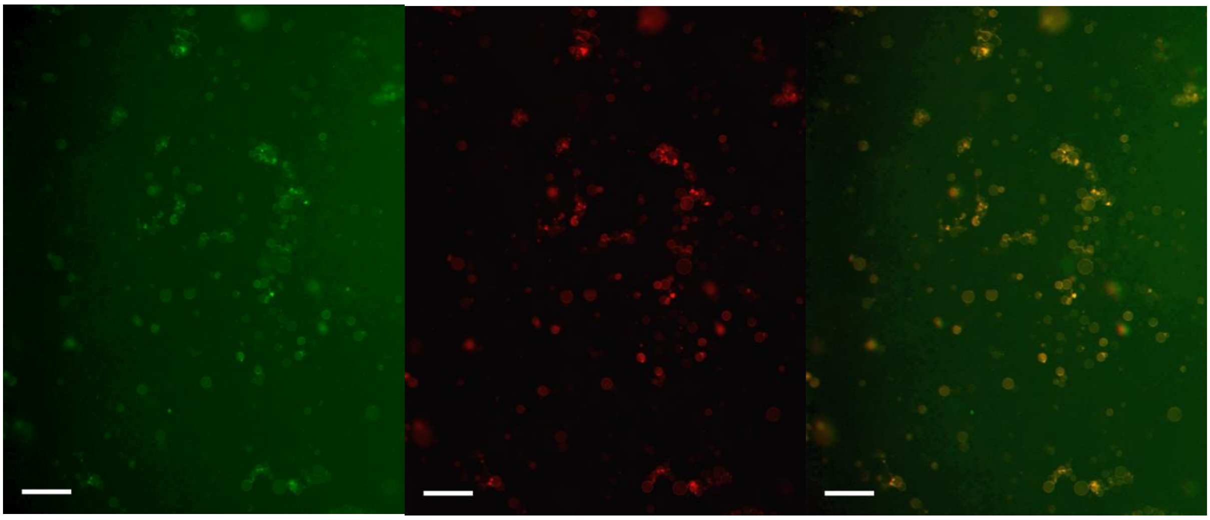

Figure S4. Protein incorporation. ATT488-derived protein signal, left; rhodamine-derived lipid signal, middle; overlay of the two images, right. The scale bar width is 20 μm.

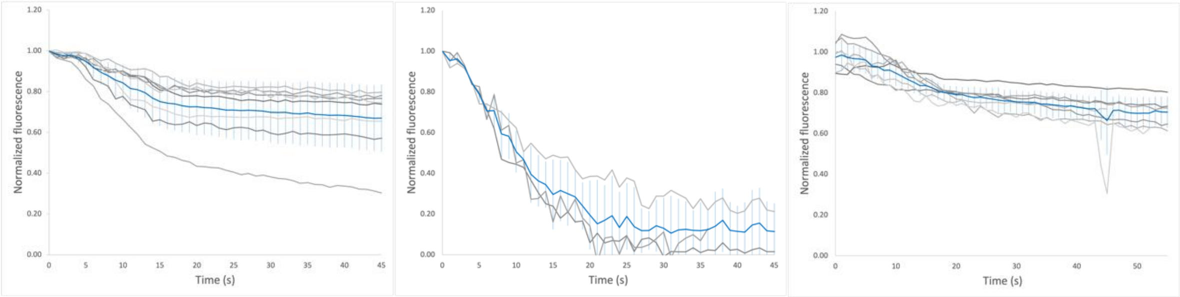

Figure S5. Individual flux curves from Fig. 3. No protein control, left; PcoB, middle, E255A mutant, right. The individual vesicles are shown in shades of gray, and the average in blue. The standard deviation shown as blue bars on the average curve.

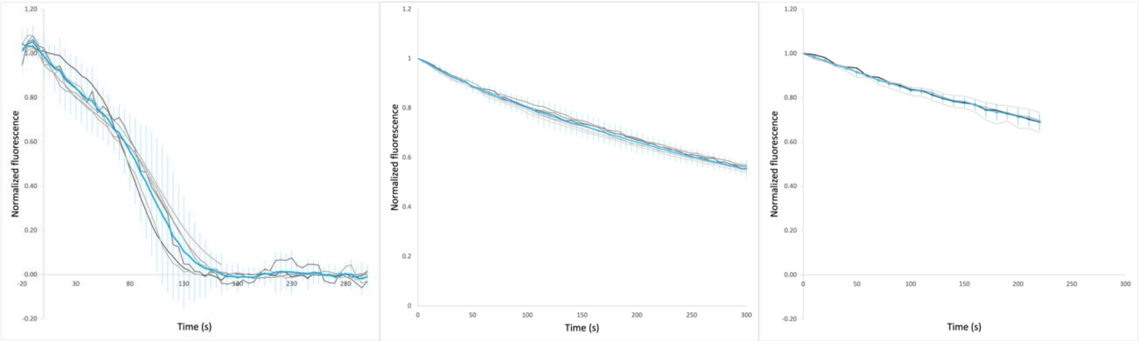

Figure S6. Individual flux curves from Fig. 4. 1 mM Cu, left; 0.5 mM Cu, middle; 1 mM Cu, no protein, right. As in Fig. S5, the individual curves are shown as shades of gray, and the average in blue.
